# Small extrachromosomal circular DNAs, microDNA, produce short regulatory RNAs that suppress gene expression independent of canonical promoters

**DOI:** 10.1093/nar/gkz155

**Published:** 2019-03-04

**Authors:** Teressa Paulsen, Yoshiyuki Shibata, Pankaj Kumar, Laura Dillon, Anindya Dutta

**Affiliations:** Department of Biochemistry and Molecular Genetics, University of Virginia School of Medicine, Charlottesville, VA 22908, USA

## Abstract

Interest in extrachromosomal circular DNA (eccDNA) molecules has increased recently because of their widespread presence in normal cells across every species ranging from yeast to humans, their increased levels in cancer cells and their overlap with oncogenic and drug-resistant genes. However, the majority of eccDNA (microDNA) in mammalian tissues and cell lines are too small to carry protein coding genes. We have tested functional capabilities of microDNA by creating artificial microDNA molecules mimicking known microDNA sequences and have discovered that they express functional small regulatory RNA including microRNA and novel si-like RNA. MicroDNA are transcribed *in vitro* and *in vivo* independent of a canonical promoter sequence. MicroDNA that carry miRNA genes form transcripts that are processed by the endogenous RNA-interference pathway into mature miRNA molecules, which repress a luciferase reporter gene as well as endogenous mRNA targets of the miRNA. Further, microDNA that contain sequences of exons repress the endogenous gene from which the microDNA were derived through the formation of novel si-like RNA. We also show that endogenous microDNA associate with RNA polymerases subunits, POLR2H and POLR3F. Together, these results suggest that microDNA may modulate gene expression through the production of both known and novel regulatory small RNA.

## INTRODUCTION

Extrachromosomal circular DNA (eccDNA) exists within all eukaryotic organisms tested (1–17) and come from consistent hotspots within the genome including 5′UTRs, exons and CpG islands (5–9). For a recent review see (18). The majority of eccDNA in normal cells are small in size, 200–400 base pairs, though they can range up to tens of thousands of base pairs (5,6,8,9,19). Mega-base sized eccDNA molecules have been found to amplify oncogenes, and smaller forms of eccDNA (>10,000 base pairs) have recently been found to also amplify oncogenes (20–22), drug-resistant genes (23) and tissue specific genes (5,8,19,24). The smallest types of naturally occurring eccDNA, <1000 bp, are called microDNA (5,6,8). The limited size of these molecules precludes them from carrying full protein coding gene sequences and or promoter sequences.

By electron microscopy, endogenous microDNA are both single and double stranded (5,8). We hypothesized that microDNA can be transcribed based on previous research that suggested that single-stranded circular DNA of 34–89 base pairs can be transcribed without a promoter *in vitro* and within cells by a rolling circle mechanism in which the polymerase travels around the circle 12–260 times (25–27). However these circles are much smaller than the average microDNA. *Paramecium tetraurelia*, on the other hand, creates double-stranded DNA circles of unknown length by fusing transposon-derived sequences and these circles also express small regulatory RNA (28).

Here, we investigate whether microDNA mimics are capable of being transcribed in mammalian cells without a canonical promoter and whether the transcripts are functional within a cell. Both single-stranded and double-stranded microDNA (ranging from ∼180 to 400 base pairs) are transcribed without a promoter *in vitro* and *in vivo*. RNA is produced from both strands of microDNA without strand bias. MicroDNA containing miRNA coding sequences, but without the promoter of the gene, produce functional miRNA capable of knocking down both a luciferase reporter and endogenous mRNA targets. MicroDNA are known to be enriched in genic regions (8,19,24) so that some microDNA carry exon sequences. We report that microDNA arising from exons can also affect gene expression by expressing novel si-RNA that targets the parental gene that the microDNA were derived from. We also show RNA polymerase subunits are associated with endogenous microDNA, giving further evidence that microDNA molecules can be transcribed in cells. Together these results show that microDNA could produce functional regulatory RNA, both miRNA and novel si-RNA, and suggest a new mechanism of how genomic plasticity and instability can lead to changes in gene expression.

## MATERIALS AND METHODS

### Cell culture

HCT116, 293A and 293T cells were cultured in Dulbecco’s modified Eagle’s medium (DMEM) supplemented with 10% fetal bovine serum, 100 U/ml penicillin and 100 μg/ml streptomycin in an environment containing 5% CO_2_ at 37°C. 293T cells were cultured in McCoy’s medium supplemented with 10% fetal bovine serum, 100 U/ml penicillin and 100 μg/ml streptomycin in an environment containing 5% CO_2_ at 37°C.

### Artificial microDNA synthesis

**Figure 1.**
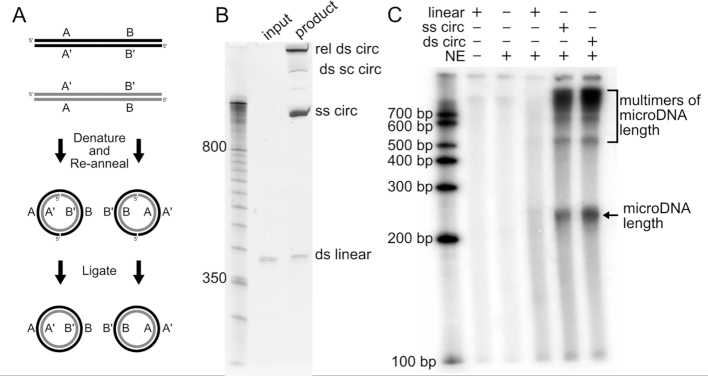
(**A**) Diagram of artificial microDNA creation by LAMA. (**B**) Circular and linear products of LAMA run on a denaturing PAGE gel before and after ligation cycles. Circular DNA accumulates to form ssDNA, nicked and supercoiled dsDNA. (**C**) 32P-UTP-labeled RNA run on PAGE gel after *in vitro* transcription assay. Products are seen at size ranges of multiples of the microDNA length. Ss: single-stranded; ds: double-stranded; sc: supercoiled; rel: relaxed or nicked; circ: circular; NE: HeLa nuclear extract. Representative replicate of duplicates.

Artificial microDNA molecules containing known microDNA sequences were created using a protocol published by Du *et al.* (29). In short, microDNA sequences were amplified out of HeLa genomic DNA using polymerase chain reaction (PCR). A circularly permuted molecule was created through PCR amplification of both complementary sequences (See figure 1), which was then cloned in the appropriate order into a pUC19 plasmid using an In Fusion HD Cloning Kit (Takara). The substrates were amplified using Phusion Polymerase PCR (NEB). The linear double-stranded molecules were denatured and renatured to produce both the parental linear molecules and a circle with nicks on each strand nearly half-way around the circle, which were then ligated by Taq Ligase (NEB). The products were taken through 10 cycles of denaturation, annealing and ligation to enrich for circular DNA. Residual linear DNA was digested using ExoI and ExoIII (NEB), and the products were separated on a denaturing PAGE gel. The band corresponding to dsDNA circles was excised and the DNA was extracted for the *in vitro* transcription reactions. Unless otherwise noted, the final products after the ligation cycles and exonuclease digestion were transfected into cells.

### *In vitro* transcription assay

The artificial microDNA (100–200 ng per 50 μl reaction) were transcribed for 4 h *in vitro* in an IVT buffer (40 mM Tris-HCl, pH 7.9, 6 mM MgCl_2_, 10 mM dithiothreitol, 2 mM spermidine, 0.1 mM NaCl), rNTPs (2 mM rATP, 2 mM rCTP, 2 mM rGTP, 0.4 mM rUTP), [α-32P]-UTP (volume dependent on radioactivity) and 20 μg of HeLa nuclear extract (Millipore) at 37°C. The radioactive product RNA was heat denatured and run on a denaturing (urea) PAGE gel. The gel image was captured using a phosphor imaging screen and a gel imager.

### Transfections of microDNA

MicroDNA were transfected in cells using Lipofectamine LTX according to the manufacturer’s instructions. RNA was isolated 24 h after transfection. Plates with 0 ng of microDNA received 100 ng of a GFP plasmid as carrier. By transfection of a GFP expressing plasmid, we estimate that 70–80% of the 293 cells take up the plasmid.

### RNA isolation and quantification

RNA was extracted using TRIZOL according to the manufacturer’s instructions (Ambion). The cDNA was created using the miScript II RT kit (QIAGEN). Specifically, the pre-microRNA sequences were quantified by creating cDNA with the miScript II RT Kit with miScript HiFlex Buffer and then amplified by QPCR with primers that flank the mature microRNA sequence within the pre-microRNA molecule. The mature microRNA sequences were quantified by creating cDNA of mature microRNA with the miScript II RT Kit with miScript HiSpec Buffer and then amplified by QPCR using a primer that targets the microRNA sequence and the 10X miScript Universal Primer. The miScript II kit was used to selectively amplify the short microRNA and not the longer product that may be created by ligation of the 3′ adaptor to pre-microRNA. QPCR was performed using Power SYBER Green Master Mix (Life Technologies).

### Luciferase assays

DNA oligonucleotides designed to carry the sequence of the mature miRNAs encoded by the microDNA (miR191, miR126, miR145) were cloned into the siCHECK (Promega) vector into the 3′UTR of the Renilla luciferase gene such that the sequence complementary to the miRNA was in the + strand. The effect of miRNA produced by microDNA on the siCHECK vector was quantified using the Dual-Luciferase Reporter Assay System (Promega), according to manufacturer’s instructions.

### Construction and infection of HaloTag vectors

The plasmid pENTR4-HaloTag that encoded the HaloTag sequence was obtained from Addgene. POLR2H and POLR3F cDNA were amplified from hORFeome V5.1 clones. PCR-amplified POLR2H or POLR3F cDNA was inserted in frame downstream of HaloTag sequence. HaloTag, HaloTag-POLR2H or HaloTag-POLR3F was subcloned into the pCW plasmid vector.

293T cells were transfected with plasmids pCW-HaloTag (or pCW-Halo-fusions), psPAX2 and pCMV-VSV-G using lipofectamine 2000. Lentivirus was harvested from the supernatant after 48 h, cleared by centrifugation and passed through a 0.45-μm filter. To obtain stably transduced 293A clonal cell derivatives expressing HaloTag fusion proteins, lentivirus was added to 293A cells in the presence of 6 μg/ml polybrene followed by selection with 3 μg/ml puromycin and isolation of clones by dilution cloning.

### HT cell lysate preparation and pull-down on Halo-Link beads

All the reagents used were pre-chilled, and the entire procedure was performed on ice. HT-fusion proteins were induced by adding 1 μg/ml doxycycline to cells in a 15-cm plate. Two days later, cells were washed with phosphate-buffered saline, scraped and transferred into a micro-centrifuge tube. After centrifugation, five packed cell volume (PCV) Hypotonic Buffer was added to cell pellets, allowing the cells to swell for 15 min on ice. Hypotonic Buffer was removed after spinning. Cells were suspended in 0.5 PCV of Buffer LS and equal volume of Buffer LS with 600 mM NaCl and 0.2% Triton X-100 was added. Cells were homogenized by passing 10 times through a 27G hypodermic needle and rotated for 20 min. After centrifugation, the supernatant was transferred into a new microcentrifuge tube, and equal volume of Buffer LS to the supernatant was added.

Cell lysate was added to the equilibrated HaloLink Resin and incubated by mixing on a tube rotator for 30 min at room temperature. After centrifugation, supernatant was saved as sample flow through. Resin was washed with PD Washing Buffer five times. The beads were boiled in Laemmli sample buffer to obtain the eluates.

### MicroDNA extraction and identification

HT or HT-POLR3F/POLR2H associated DNA was purified with QIAprep Spin Miniprep Kit according to its instruction manual and amplified by rolling circle amplification (RCA) as described (6,8).

Initially paired-end high-throughput sequencing (250 cycles PE) was performed on the RCA products, according to the manufacturer’s protocol (Illumina) on the Illumina MiSeq at the University of Virginia DNA Sciences Core (Charlottesville, VA, USA) (Table 1). Read quality was checked by program fastqc (FASTX-Toolkit) and was found that the median read quality was <28 after position 150 on the read. Therefore to remove the bad quality bases we made each library 150 PE and did the downstream analysis. We used Burrows–Wheeler Aligner with maximal exact matches (BWA-MEM) to align the reads to the human hg38 genome, allowing for split reads under default conditions. Like our previous publication (5,6), we used split reads mapped position to identify microDNA coordinate at base pair resolution. In summary to identify microDNA we consider paired-end reads that had one end mapping uniquely to the reference genome (mapped end) and the other end not mapping to the reference genome continuously, but coming from a split-read (the junctional read). Furthermore, the two parts of the split read have to flank the linked mapped read. The identified microDNA would represent genomic coordinates of potential microDNA junctions created by the ligation of two ends of a linear DNA. In addition to this, we also check polarity (strand information) of both the split read (should be map in the same orientation) and mapped read in pair (this should be opposite to split read).

**Table 1.** MicroDNA diversity associated with HT, and HT fused with indicated RNA polymerase subunits

Sample name	Paired-end reads	Mapped reads	MicroDNA	Mean (random 500K PE mapped reads)	SD
2H-R1	3 182 235	3 104 725	54 592	16 531	94
3F-R1	1 479 615	1 434 156	19 217	9675	60
HT-R1	11 472 991	9 152 503	14 882	2473	42
2H-R1	10 314 411	10 081 812	112 572	17 072	110
3F-R1	2 868 822	2 780 519	30 038	9751	66
HT-R1	1 092 336	854 021	3732	2842	18

To normalize for the number of paired end reads in the three libraries, 500 000 mapped reads were randomly selected from each library and the number of unique microDNAs in the samples determined. This was done 10 times and the mean microDNA number and their standard deviation indicated in the last two columns.

Summary of reads obtained from HT, HT-POLR2H and POLR3F associated microDNA libraries (two independent sequencing runs done on separate days, R1 and R2). Low quality bases from the 3′ end of reads were removed by making 150 (read length) PE reads from 250 (length) PE reads. We also made 75 bases PE reads from the same library to identify microDNA shorter than 150 bp. Total number of unique microDNA (unique microDNA junctions) identified from each run is indicated.

### Evaluation of complexity of microDNA associated with RNA polymerases

The microDNA were identified from the HaLo-Tag pull-downs as above. Because of the differences in number of mapped reads obtained from the HT-, HT-POL2H- and HT-POL3F-associated microDNA, we used a random sampling approach to compare the microDNA complexity among samples (Table 1). The same number of mapped reads was randomly extracted from the HT-POLR2H and HT-POLR3F libraries, as the total number of mapped reads in the HT library. This was done 10 times. Each randomly selected set of reads was processed as above and the number of unique microDNAs identified counted (Table 1). The mean and standard deviation of the 10 samples is presented in Figure 6C.

## RESULTS

### Synthesis of microDNA mimics

We created synthetic microDNA mimicking known microDNA sequences by utilizing a technique, called ligase-assisted mini-circle accumulation (LAMA), which relies on cycles of annealing, ligation and denaturation to produce small DNA circular molecules (Figure 1A) (29). Utilizing sequencing data of microDNA isolated from human cancer cell lines, we designed circles that mimic known microDNA overlapping with microRNA sequences or with exons of non-coding or protein coding genes. Both single-stranded and double-stranded artificial microDNA molecules were created and isolated (Figure 1B). The sequences of the circular molecules created are listed in Supplementary Table S1. Double strandedness of specific isoforms was verified by digestion with restriction endonuclease, while topoisomerase I was used to distinguish supercoiled from relaxed circles (Supplementary Figure S1).

### MicroDNA mimics are transcribed *in vitro*

To test whether microDNA are transcribed we used an *in vitro* transcription system using HeLa nuclear extract that contains human RNA polymerases. We isolated the single-stranded and double-stranded microDNA (containing the hsa-mir-145 sequence) after separation on the denaturing PAGE and added transcriptionally competent HeLa nuclear extract, rNTPs and radiolabeled rUTP. Both single-stranded and double-stranded circular microDNA molecules are transcribed but the linear DNA control containing the same sequence is not transcribed (Figure 1C). Relaxed circles produced significantly more transcripts than supercoiled circles (Supplementary Figure S1). The *in vitro* transcription assay experiment was repeated with two other microDNA sequences (microDNA carrying hsa-mir-126 sequence and the hsa-let-7a sequence) and similar results were found (Supplementary Figure S2). Each *in vitro* transcription experiment was validated with a second replicate. The RNA products show distinct lengths that correspond to multiples of the microDNA sequence length. This suggests that some RNA polymerases fall off the DNA template after going around the circle once and some continue around the circle multiple times.

Because the microDNA sequences did not contain known promoters, this result also shows that the short double-stranded DNA circles, as well as the single-stranded DNA circles, are transcribed independent of a canonical promoter sequence by human RNA polymerases, whereas a linear DNA fragment is not transcribed. We hypothesize that the bending of the double-stranded DNA enables the binding of TATA-binding proteins (30–33) to recruit RNA polymerase that initiates transcription independent of a canonical promoter.

To determine where transcription initiates on a microDNA mimic (microDNA with hsa-mir-191 sequence), we performed an *in vitro* transcription reaction and then quantified the RNA arising from different regions of the microDNA sequence. We found that the transcription is not uniformly distributed around the circle, but has some sequence bias (Supplementary Figure S3). This suggests that certain sequences within the microDNA are more likely to be bound by transcription initiation machinery than others.

### MicroDNA mimics are transcribed *in vivo*

**Figure 2.**
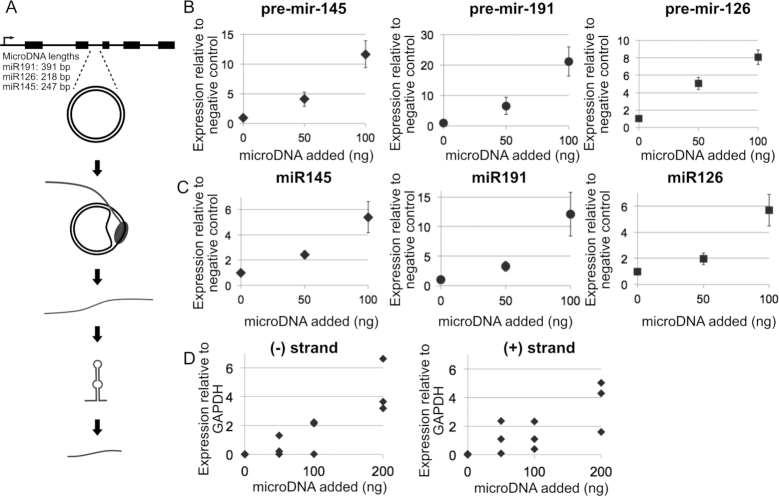
(**A**) Diagram of transcription of microDNA carrying only the promoterless pre-microRNA part of the microRNA gene. The transcripts are processed by endogenous RNA interference proteins into functional mature miRNA (**B**) Expression of pre-microRNA molecules after the addition of indicated amounts of the corresponding artificial microDNA molecules. Expression is relative to β-actin (hsa-mir-191 and hsa-mir-126) and GAPDH (hsa-mir-145) and normalized again to the negative control of a transfected GFP plasmid. Mean and S.E. of three transfections. (**C**) Expression of processed mature miRNA molecules after transfection of indicated amounts of artificial microDNA molecules. Expression is relative to β-actin (hsa-mir-191 and hsa-mir-126) and GAPDH (hsa-mir-145) normalized to a negative control of a transfected GFP plasmid. Mean and S.E. of three transfections. (**D**) Expression of the + and the – strand of the pre-microRNA, hsa-mir-145, from microDNA molecules relative to GAPDH. Strand-specific primers were used for the reverse-transcription to form cDNA specifically from the (+) or (-) strand of RNA. Results from three transfections.

We next wanted to test whether the artificial microDNA molecules can be transcribed *in vivo*. Because some of the microDNA molecules that have been sequenced from human cancer cells contain miRNA sequences (8), we hypothesized that microDNA may be capable of expressing miRNA (Figure 2A). Artificial microDNA molecules were made that carry miRNA sequences observed in naturally occurring microDNA sequences: hsa-mir-145, hsa-mir-191, hsa-mir-126. The sequences contained only the pre-miRNA part of the gene but not the rest of the primary miRNA, and hence excluded the promoter that is found at the 5′ end of the long primary miRNA, often tens of kb away from the mature miRNA. As the amounts of artificial microDNA are increased, the levels of each of the pre-miRNA transcripts increased by 6- to 30-fold (Figure 2B). At least some of pre-miRNA transcripts are processed into mature miRNAs that are also increased concurrently by 3- to 6-fold (Figure 2C).

Additionally, the transfection of linear DNA carrying the same sequence as the microDNA does not increase the RNA levels confirming that the DNA must be circularized to be transcribed (Supplementary Figure S4A). To further show that the RNA is arising from circular DNA and not linear contaminants or the genomic sequence, we quantified the RNA using primers that amplify the junction sequence. We see an increase in the RNA spanning the junction as more artificial microDNA are added (Supplementary Figure S4B). The fold increase of the junctional RNA appears significantly higher relative to fold-induction seen with primers known to target the pre-microRNA within the microDNA. We believe this is because there is no endogenous RNA in untransfected cells that spans the junction of the circle. Therefore, the fold-induction using the primers spanning the junction sequence gives a more accurate representation of the quantity of RNA arising from microDNA, because there is no endogenous junction-spanning RNA elevating the basal level in untransfected cells.

Because the microDNA are transcribed without a canonical promoter sequence, we wondered whether the RNA is equally transcribed from both strands of the microDNA when double-stranded circular microDNA are transfected. Reverse-transcription with strand-specific primers and Q-PCR demonstrated that the microDNA carrying hsa-mir-145 were transcribed relatively equally from either strand (Figure 2D). The relative stabilities of the RNAs arising from the two strands and whether the RNAs are processed by RNA capping and poly-A addition is unknown and will require further research.

Overall, these results suggest that microDNA like molecules can be actively transcribed within cells. The specific character of microDNA allowing for its relative independence from a canonical promoter requires further determination. This research gives insight into how these circular DNA molecules, till now assumed to be inert byproducts of DNA metabolism, could contribute to cell physiology by actively forming RNA transcripts.

### MicroRNAs produced from microDNA mimics are functional

**Figure 3.**
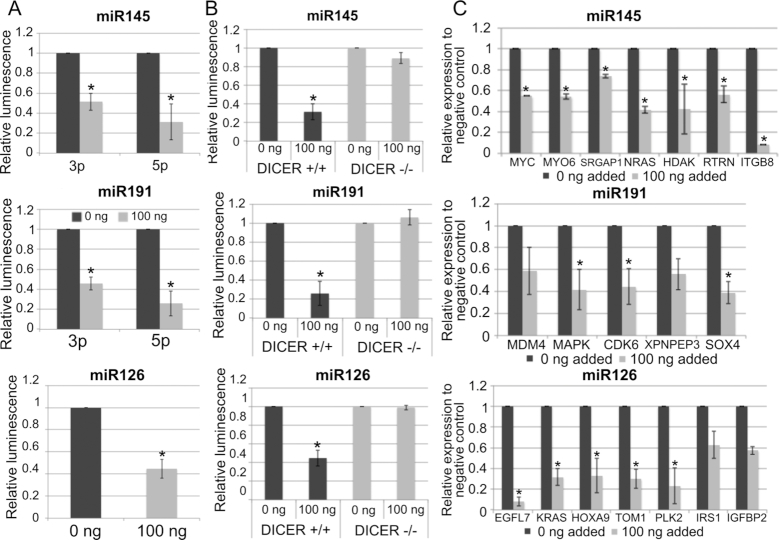
(**A**) Transfection of artificial microDNA, carrying indicated pre-miRNA sequences, to 293T cells decreases expression of a co-transfected Renilla luciferase reporter containing a sequence complementary to the miRNA sequence within its 3′ UTR. RL activity expressed relative to a co-transfected firefly luciferase and normalized again to the level in cells transfected with 0 ng of microDNA. Mean and S.E. of three experiments. * indicated *P* < 0.05 in a Student’s *t-*test. (**B**) Repression of luciferase in dual-luciferase assay is observed in WT 293T cells but not in DICER1-/- 293T cells. Mean and S.E. of three experiments. * indicated P < 0.05 in a Student’s t-test. (**C**) Endogenous cellular targets of indicated miRNAs are repressed after transfection of the synthetic microDNA carrying the indicated pre-miRNA genes. mRNAs quantitated by QRT-PCR and expressed relative to the β-actin gene and normalized to the level in cells transfected with 0 ng microDNA. Mean and S.E. of three experiments.

To determine whether microDNA produced functional miRNAs, luciferase reporters containing target sequences complementary to the miRNAs in their 3′ UTRs were co-transfected with the microDNA for dual-luciferase assays in 293T cells. Each of the microDNA molecules (containing hsa-mir-145, hsa-mir-191 or hsa-mir-126 sequences) repressed the luciferase reporter carrying the target sequence of the miRNA by >50% after transfection (Figure 3A). Further, the microDNA containing hsa-mir-145 and hsa-mir-191, which carry both the 3p and 5p sequences of the microRNA, are able to repress a luciferase reporter that contains either the 3p or 5p sequence. This shows that the transcripts arising from the microDNA form both mature sequences and function in the same manner as endogenous microRNA.

We next examined whether the repression of a luciferase reporter by a co-transfected microDNA is dependent on the endogenous RNA interference pathway. The introduction of each microDNA carrying microRNA sequences did not repress the luciferase reporter when transfected into *DICER1* KO 293T cells (34) (Figure 3B). This shows that the transcripts from microDNA need to be processed by Dicer through the same pathways as pre-miRNA produced from a chromosomal locus.

The microDNA carrying miRNA sequences also repress endogenous cellular genes that are targets of the encoded miRNA. The predicted targets were obtained from Targetscan. The microDNA carrying hsa-mir-145 sequence repressed mir-145 targets by up to 40%; hsa-mir-191 microDNA repressed mir-191 targets by up to 60%; hsa-mir-126 microDNA repressed downstream targets by up to 90% (Figure 3C). In each case, most targets were repressed to similar levels. Further, the gene repression caused by microDNA was specific to the targets of the miRNA encoded by the microDNA: for example, targets of miR-145 or miR-126 were not repressed by the introduction of microDNA containing miR-191 (Supplementary Figure S4C). Together these results show that the microDNA are potentially capable of contributing to the population of functional short RNA within a cell to influence the expression of endogenous genes.

### MicroDNA mimics containing exon sequences can repress host genes

**Figure 4.**
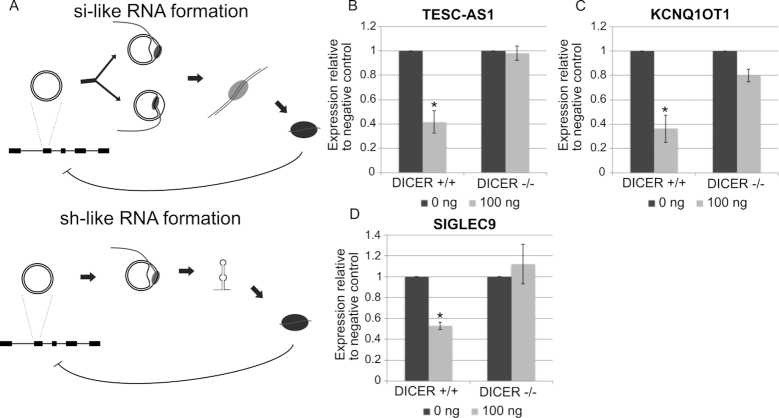
(**A**) Diagram of the theoretical mechanism of the formation of si-like or sh-like RNA from microDNA containing an exonic sequence either from transcription of both strands of the microDNA or from folding of the transcript into a short-hairpin. (**B**) Transfection of microDNA containing the sequence of exon 2 of the TESC-AS1 gene represses the expression of the TESC-AS1 gene in 293Tcells. The repression is observed in 293T cells but not in DICER-/- 293T cells. The same is seen with microDNA carrying a portion of (**C**) Exon 1 of KCNQ1OT1 and (**D**) the Exon 6 of SIGLEC9. The RNAs were quantitated by QRT-PCR, and the values expressed relative to β-actin and normalized to the cells with 0 ng of transfected microDNA. Mean and S.E. of three experiments. *P < 0.05 in a Student’s *t*-test.

MicroDNA are enriched from genes and often contain exons (8). Short hairpin RNA (shRNA) sequences have long been known to be processed into siRNAs that repress target genes, suggesting that the gene from which a microDNA are derived could be repressed by the microDNA. Alternatively, if microDNA are transcribed from both DNA strands the resulting double-stranded RNA could also be processed to a functional si-RNA (Figure 4A). Indeed, a similar phenomenon has been suggested in *P. tetraurelia* where transposon-derived DNA sequences are ligated to form circles (of unknown size) that produce siRNAs that repress transposon expression (28).

To test whether microDNA could repress the parental gene, we created artificial microDNA containing exonic sequences that we had identified in human cancer cells (Supplementary Table S1). These microDNA were transfected into 293T cells, where we get nearly 80% of the cells taking up the transfected DNA (data not shown). Three microDNA containing different exon sequences specifically repressed the expression of the host gene that contained the matching exon sequence. MicroDNA carrying the full sequence of exon 2 of the *TESC-AS1* gene repressed the TESC-AS1 mRNA by ∼60% (Figure 4B). MicroDNA encoding a portion exon of the *KCNQ1OT1* gene repressed the KCNQ1OT1 mRNA by ∼60% (Figure 4C). MicroDNA encoding the full exon 6 of *SIGLEC9* gene repressed of the SIGLEC9 mRNA by ∼50% (Figure 4D). Here again the repression of the endogenous gene by the microDNA is dependent on the RNA interference pathway, specifically *DICER1*, with the repression of TESC-AS1, KCNQ1OT1 or SIGLEC9 mRNAs significantly attenuated in *DICER1* KO 293T cells (Figure 4B–D). The expression of genes not containing homology to the microDNA mimic were not affected by the introduction of microDNA: for example, the microDNA from *KCNQ1OT1* did not repress *TESC-AS1* or *SIGLEC9* (Supplementary Figure S4D). This shows that the microDNA can also produce novel si-like RNA when the microDNA are derived from exonic sequences.

Thus, regulatory short RNAs can be produced not only from microDNA that contain pre-microRNA genes but also from microDNA that overlap with exons. This greatly expands the proportion of eccDNA now expected to contribute to gene expression changes.

### Endogenous microDNA are associated with RNA polymerases

**Figure 5.**
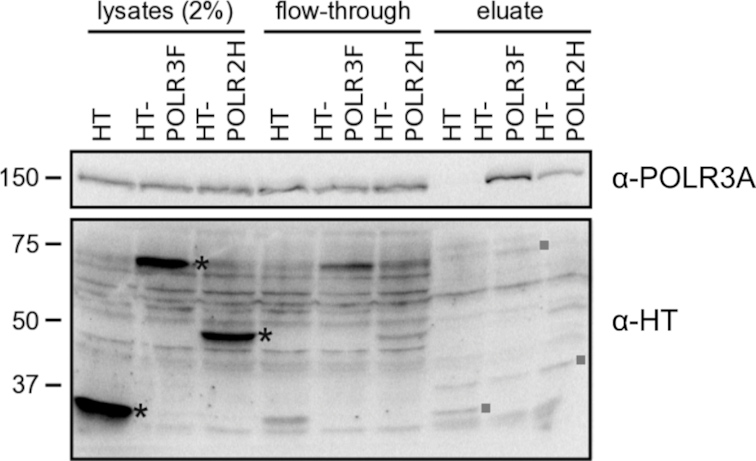
Efficiency of pull-down of RNA polymerase complex by HT. Detection of RNA polymerase III catalytic subunit (POLR3A) in the HT-POLR2H or HT-POLR3F pull-down. HaloTag-fused RNA polymerase subunit POLR2H or POLR3F was expressed in 293A cells (asterisk). The HT-tagged polymerase subunits were covalently bound to the HaloLink resin, and the non-covalently associated proteins were eluted by boiling in Laemmli Sample buffer. The HT-proteins are covalently bound to the beads and are mostly not eluted. Some covalent bonds break to release traces of the HT protein in the eluate (squares). However, non-covalently associated POLR3A of the RNA polymerase complex is specifically released in the eluates from the HT-POLR3F and HT-POLR2H pull-downs.

**Figure 6.**
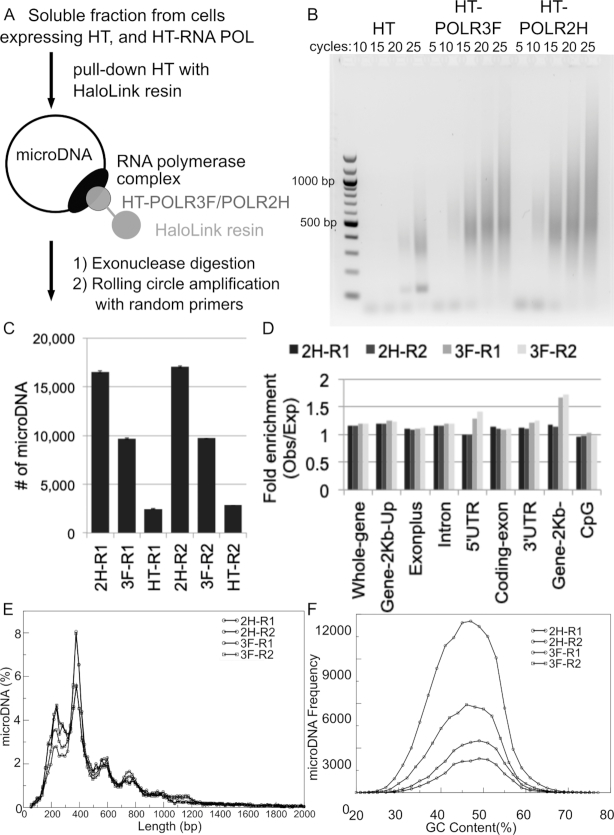
Subunits of PolI, PolII and PolIII bind to microDNA. (**A**) Diagram of pull-down of Halo-tagged RNA polymerase subunits and purification of associated microDNA for rolling circle amplification with random hexamers (RCA). (**B**) RCA products were sheared, ligated to library primers for high-throughput sequencing, amplified by PCR for the indicated cycles and comparable aliquots run on a gel and visualized by ethidium bromide fluorescence. (**C**) Complexity of microDNA in the libraries prepared from the POLR3F and POLR2H pull-downs relative to the tag-only control as measured in Table 1. The error bars indicate the S.D. from 10 random subsamples from each library. (**D–F**) Characterization of microDNA molecules pulled down by POLR3F and POLR2H. (D) Enrichment relative to random expectation of the microDNA from areas of the genome with indicated genomic features, (E) length distribution and (F) GC content.

We next tested whether RNA polymerases bind to endogenous microDNA. Epitope-tagged (Halo Tag (HT)) POLR3F (subunit of RNA Polymerase III) or POLR2H (subunit of all three RNA Polymerases) was induced by doxycycline, in 293A cells (Figure 5). The epitope tagged subunits were then captured by covalent linkage to HaloLink resin and the pull-down confirmed by western blotting of the eluate for a non-covalently associated RNA polymerase subunit, POLR3A (Figure 5). The DNA that was associated with the HT alone or HT-POL subunits was isolated, digested by exonucleases, and then amplified by multiple displacement amplification with random hexamers (Figure 6A). The amplified DNA was quantitated and found to be significantly more in the POLR3F and POLR2H eluates than in the HT alone negative control precipitates: undetectable (below limit of detection) for the HT control, 728 ng for HT-POLR3F and 408 ng for HT-POLR2H. Note that the HT alone is 33 kDa in size, and so a significant sized protein is being pulled down on the negative control HT beads. Further, PCR of the sheared RCA products ligated to sequencing adapters show that POLR3F and HT-POLR2H pull-down 2e10 (or ∼1000) fold more DNA than the HT control (Figure 6B). Because POLR2H and POLR3F both bind to endogenous microDNA, it suggests that microDNA could be transcribed by RNA Polymerase III, and possibly RNA Polymerase I and II.

The rolling circle amplification products were subjected to Illumina sequencing to identify the microDNA and determine the number of unique sites in the genome represented in the microDNA library (complexity). The HT-POLR3F and HT-POLR2H associated libraries yielded 27- and 6-fold more complexity than the HT-associated library (Table 1). Since the yield of DNA after rolling circle amplification was significantly more in the two POLR precipitates, we also compared the complexity by randomly sampling equal numbers of high-throughput reads from the three precipitates. Even after equalizing read numbers, the HT-POLR3F and HT-POLR2H precipitates had microDNA at 3- to 8-fold higher complexity than that associated with just the HaloTag (Table 1 and Figure 6C), providing further evidence that microDNA bind to RNA polymerase subunits. Sequencing of the multiple displacement amplification products also confirmed that the microDNA associated with the RNA Polymerases have the same characteristics as found in our previous studies including length peaks at around 200–400 base pairs with a periodicity of nucleosome length DNA (Figure 6E), and GC content around 45–50% (Figure 6F). This shows that naturally occurring microDNA are associated with RNA polymerases, consistent with the hypothesis that they can be transcribed to produce functional regulatory small RNAs.

We have reported before that the microDNA in mammalian cells are 4–10X enriched relative to random expectation from 5′ UTRs, exons, genes and CpG islands (8). In contrast, the RNA polymerase-associated microDNA are more uniformly distributed throughout the genome (Figure 6D), suggesting that the RNA polymerase subunits have a lower affinity for microDNA from gene or CpG island-derived areas.

## DISCUSSION

In summary, we have found that microDNA mimic molecules within cells are capable of being transcribed without a canonical promoter to form functional microRNA and novel sh- or si-RNA. The transcripts from microDNA mimics that carry microRNA sequences are processed into mature microRNA that can repress expression of downstream targets and a luciferase reporter. MicroDNA arise from about 5% of the genome in the chicken DT40 cell line and 0.4–1.5% of human HeLa, C4-2 and LNCap cell lines (Supplementary Figure S5), and are enriched from genic regions (5,6,8). An intriguing finding of this study is that microDNA mimics that carry exonic sequences form novel sh- or si-RNA that represses the gene from which it originated. Additionally, subunits within RNA polymerase complexes (POL2H and POL3F) are bound to naturally occurring microDNA, adding to the possibility that endogenous microDNA are transcribed. Collectively, these results support the hypothesis that microDNA could be functional in cells, actively repressing genes through the RNA interference pathway by producing microRNA and novel si-RNA.

EccDNAs, including microDNA, are found to be significantly increased in cancer cells (5,6,8,21). We know that long eccDNA (>10,000 kb) can amplify genes, including oncogenes, in cancer and change gene expression patterns that contribute to oncogenesis. Our current results suggest that the microDNA could similarly regulate gene expression patterns through the formation of novel regulatory RNAs arising from microDNA.

The length distribution of eccDNA in various tissues and organisms has a distinct pattern of peaks that correspond to lengths of DNA bound by nucleosomes suggesting that the excision of DNA from the genome may be regulated by nucleosomes. Interestingly, it has recently been discovered that artificial eccDNA molecules introduced into cancer cell lines have high stability but are quickly transcriptionally silenced by epigenetic mechanisms (35). Although 359-bp long microDNA like plasmids have been shown to be assembled *in vitro* into mono- or di-nucleosomes (36), it is unclear whether the microDNA in cells are chromatinized, and if they are, whether they can be epigenetically repressed like the long eccDNA. Therefore, it will be interesting to test in the future whether the microDNA can or cannot be epigenetically regulated.

Because the the correspondence of the length of the microDNA with nucleosomes, it has been suggested that microDNA may arise at least partially as a byproduct of apoptosis (9). Even in this study, however, the authors noted that microDNA arise from specific parts of the genome and that they arise in lymphoblasts that are resistant to the apoptosis-inducing agents methotrexate or l-asparaginase and in cells not treated with any apoptosis-inducing agents so that the authors qualified their suggestion by saying that apoptosis is not the only source of microDNA. Our results that microDNA formation is diminished in DT40 cells mutant for the mismatch repair gene *MSH3* (8), and the specific association of endogenous microDNA with RNA polymerase subunits that we report here reinforces the suggestion that microDNA are not solely produced by apoptosis, if at all.

More research is necessary to determine the exact mechanism by which microDNA are transcribed, but it is clear that the circularization is important, because the equivalent linear DNA was not transcribed *in vitro* (Figure 1C and Supplementary Figure S2) or *in vivo* (Supplementary Figure S4A). Transcription of microDNA without the requirement of a canonical promoter sequence may occur because of structural features unique to the microDNA that attract an RNA polymerase, or because cryptic promoters as seen in (37) are created by the circularization of the linear DNA into microDNA. At most, 1 or 2 nucleosomes could be assembled on circles of 359 bp *in vitro* (36,38). Thus, abnormal chromatinization may leave the intervening naked DNA more accessible to transcription factors including RNA polymerases (39) allowing promoters or cryptic promoters to be more readily bound. The small size of the microDNA molecule may also contribute to its spontaneous transcription through the formation of flipped bases (40) and bubbles of ssDNA (41), the latter known to initiate transcription (42). Further, the bent shape of the small circular DNA itself may signal for the binding of TATA-binding proteins (30–33), though the bent shape of DNA can also limit its transcription when associated with a *bona fide* promoter (43). Lastly, it has also been shown that nicks can initiate transcription that suggests that if microDNA molecules contain nicks then that could also contribute to their transcription (44).

We have previously reported that microDNA arise from epigenetically active gene-rich areas within the genome (8). Therefore, transcription can lead to microDNA formation, which could lead to repression of the parent gene by generating regulatory short RNAs. This could be a negative feedback mechanism that represses GC-rich genes when they are transcribed at a high level and produce microDNA, most likely from some DNA repair process. It is intriguing that a similar pathway has been proposed for the silencing of transposon genes in the germline nucleus of *P. tetraurelia* where transposon-derived DNA sequences are ligated to form circles (of unknown size) that produce siRNAs that associate with PIWI proteins to repress transposon expression (28).

One criticism of our study is that the amount of synthetic microDNA we transfected into cells to see increase in microDNA-encoded regulatory RNAs is significantly higher than endogenous levels of microDNA. We believe that this is because of inefficiencies introduced by several factors: the presence of endogenous microRNA that sets a high basal level of the RNA for comparison, the low number of DNA molecules taken up per cell and the possible saturation of endogenous RNA polymerases. The microDNA sequences selected for this study were chosen because of the existing knowledge of their encoded microRNAs and their target genes. As a result, the fold-increase of microRNAs arising from transfected microDNA is diminished by the high basal level of endogenous microRNA in the untransfected cell. Indeed, when we look for microDNA-junction-specific RNAs, the fold-change is very high because there is very little endogenous RNA to set a high basal level (Supplementary Figure S4B). Another important factor is that we do not know how much of the added microDNA is being taken up by the cells. It is very likely that these fractions/numbers are low leading to an under-representation of the effect of the synthetic exogenous microDNA. In addition, the microDNA molecules have no inherent structural feature that would cause them to escape from the endo-phagosome and be transported into the nucleus therefore limiting the amounts of the transfected microDNA that can be transcribed. Finally, it is unknown if there is sufficient free RNA polymerase to associate with the newly arriving nuclear microDNA, further decreasing the proportion of exogenous microDNA that are actively transcribed. Overall, we believe the effect of the microDNA transfected into the cells is under-represented by our experiments because of these limiting factors.

The controls in all experiments were designed so that the only variant between experiment and control is the specific sequence of the artificial microDNA transfected into the cell. This ensures that the differences in expression of the genes measured by QRT-PCR and luciferase assays can be attributed to the introduction of the exogenous microDNA. Further, the results are robust because each experiment was repeated multiple times and produced consistent results.

In summary, inspired by the prevalence of small eccDNA (microDNA) in normal and cancer cells, we examined whether mimicking molecules could express functional gene products. The results suggest that microDNA could express regulatory short RNAs, raising the possibility that microDNA could cause changes in cell phenotype by regulating gene expression.

## Supplementary Material

Supplementary Data

## Notes

Present address: Anindya Dutta, Department of Biochemistry and Molecular Genetics, University of Virginia School of Medicine, Charlottesville, VA 22908, USA.
