# Supplementary material for: Small extrachromosomal circular DNAs, microDNA, produce short regulatory RNAs that suppress gene expression independent of canonical promoters
Source: Nucleic Acids Res. 2019 Mar 4;47(9):4586–96. doi: 10.1093/nar/gkz155 (PMC6511871; doi:10.1093/nar/gkz155)
Supplement: Supplementary Data [file gkz155_supplemental_file.pdf]

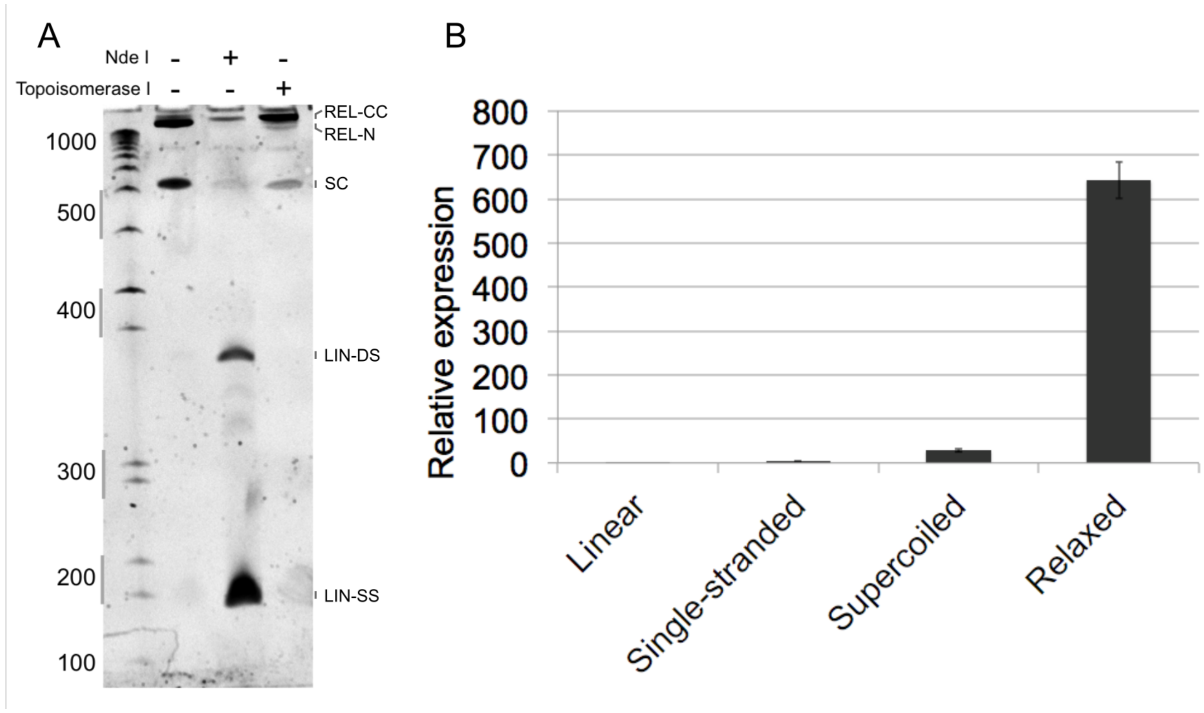

Supplementary Figure 1 Circular microDNA mimic molecules of all topologies are transcribed by RNA polymerases in HeLa nuclear extract *in vitro* transcription assay. (A) Different topologies of microDNA (with KCNQ1OT1 sequence as example) analyzed by incubation with NdeI digestion (cuts the double-stranded microDNA twice) or topoisomerase (relaxes supercoiled microDNA). REL-CC: Relaxed covalently closed circular DNA; REL-N: Relaxed nicked circular DNA; SC: Supercoiled circular DNA; LIN-DS: Linear double stranded DNA; LIN-SS: Linear single stranded DNA. Representative replicate of duplicates. (B) QPCR of RNA products obtained by IVT of microDNA with indicated topologies or strandedness (with hsa-mir-191 sequence) Mean and S.D. of 3 replicates.

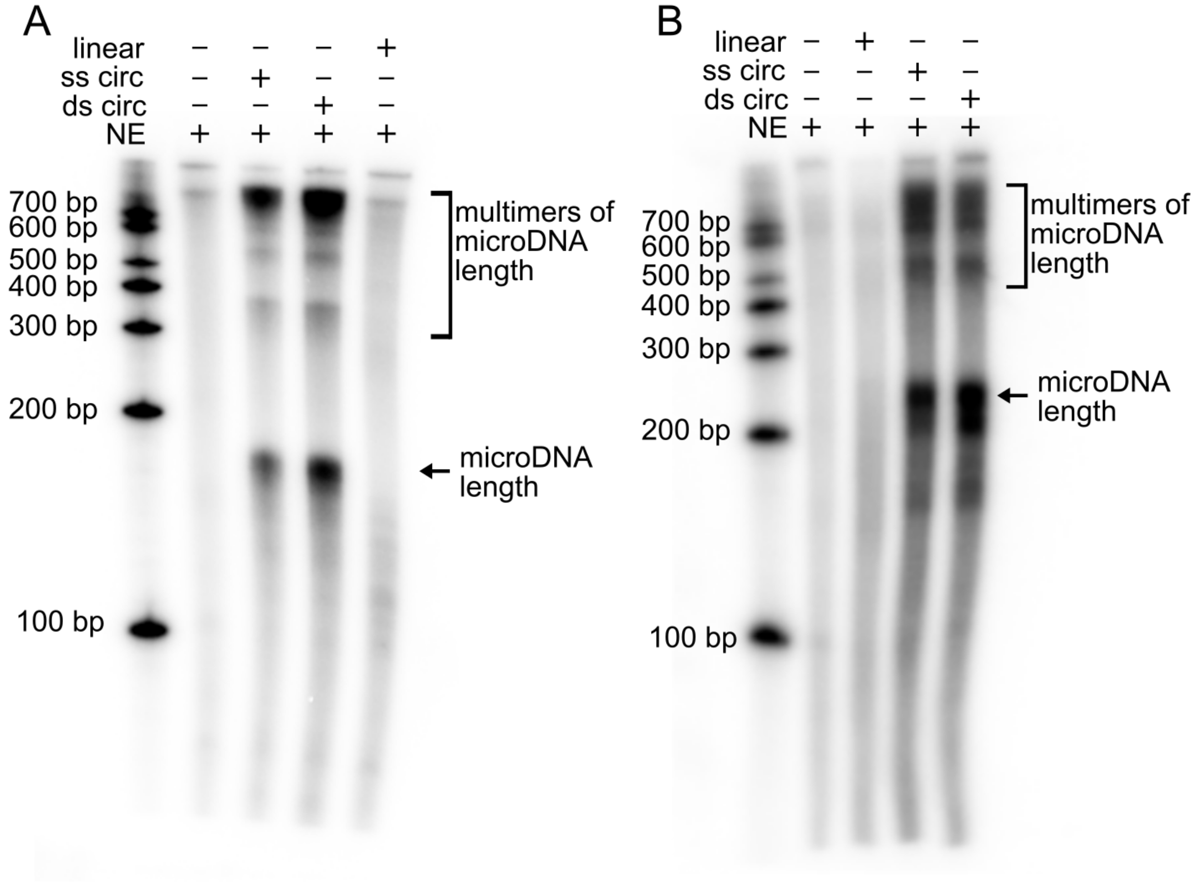

Supplementary Figure 2 The in vitro transcription of microDNA by RNA polymerases within HeLa Nuclear Extract is verified with two other microDNA sequences: (A) microDNA containing hsa-let-7a (B) microDNA containing hsa-mir-145. Representative replicate of duplicates.

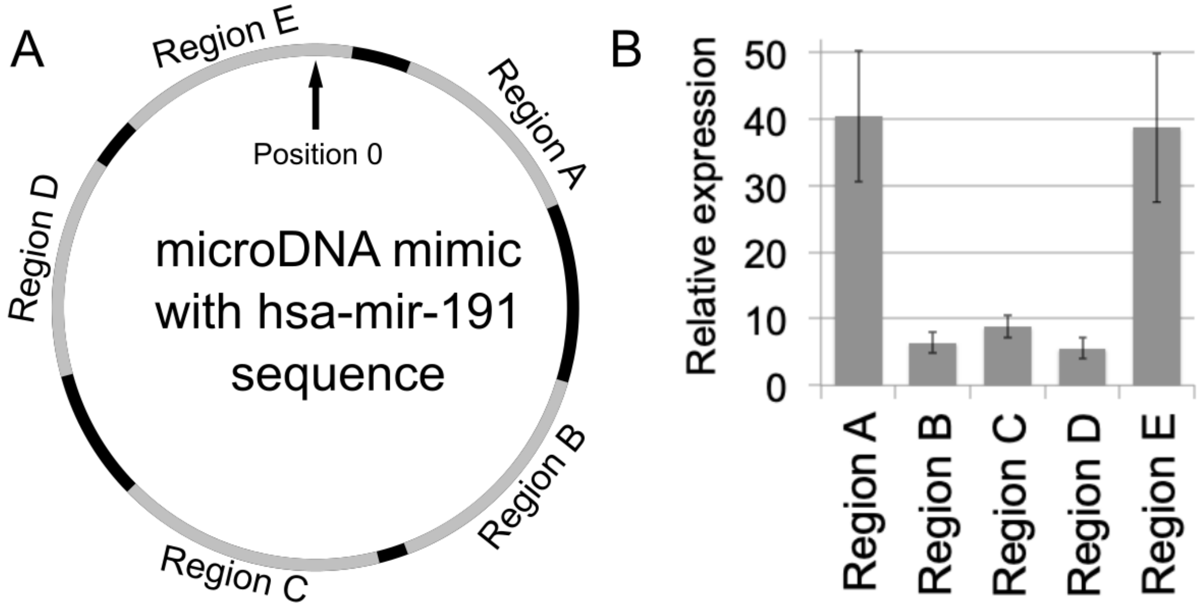

C DNA sequence of each region:

A:TTTAGCGAGATGCGGGCTGAGGCAGCCCTGGGGGAGTCACTACCATTGC

B:CTCCCCAGGGTTCCAGAGATGGCCACCAGCCTCGGCTCTCCCACGTCTACAC

C:CCTGAGGGCGCAACTGAGAGGAACTGAGACCCAAGCAGCTCAGTAAAGTATGTC

D:AGGAGAGCAGGGGACGAAATCCAAGCGCAGCTGGAATGCTCTGGAGACAACAG

E:TTCCGTTGCCCGCTGTCCAGCCGTTGGCGGGGGGCGGGGGAGCTCCTGCCCCC

Supplementary Figure 3 Distribution of transcripts from microDNA is not random (A) Diagram of regions of the microDNA whose transcripts are quantitated by Q-RT-PCR. (B) Quantification of RNA arising from each region of the microDNA after normalizing to efficiency of the primer pairs when they are used to amplify the circular DNA. Mean and S.E. of 3 replicates. (C) Sequence of microDNA within each specified region

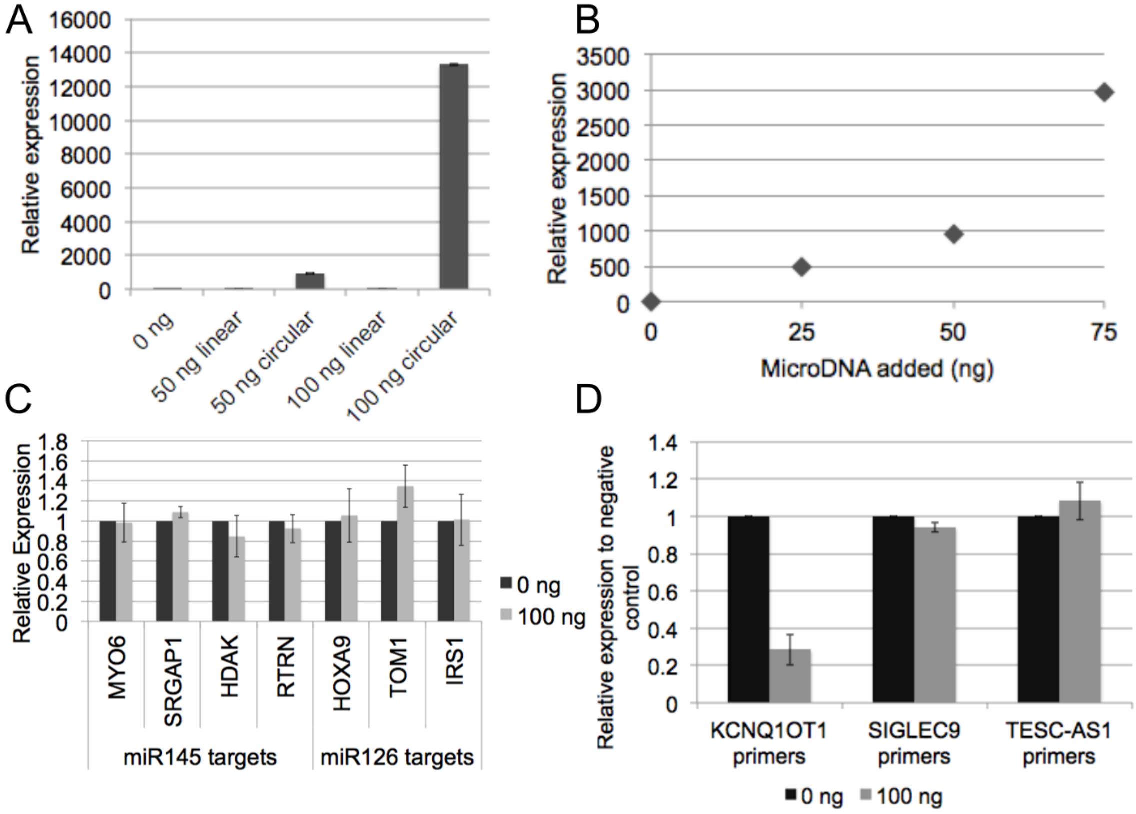

Supplementary Figure 4 RNA transcribed from circular microDNA specifically and the repression of gene expression by RNA transcripts arising from microDNA is sequence-specific. (A) Circular, but not linear, molecules of the hsa-mir-126 microDNA increases the encoded RNA when transfected into 293T cancer cells. Mean and S.D. of 3 replicates. (B) QPCR of RNA arising only from microDNA quantified using primers which amplify the junction sequence of the microDNA (containing hsa-mir-126) Mean and S.D. of 3 replicates. (C) When the microDNA containing the miR-191 sequence was transfected into human cancer cells, genes which are not targeted by miR-191 were not repressed. Mean and S.D. of 3 replicates. (D) When the microDNA containing the KCNQ1OT1 microDNA sequence was transfected into human cancer cells, genes other than KCNQ1OT1 were not repressed. Mean and S.D. of 3 replicates.

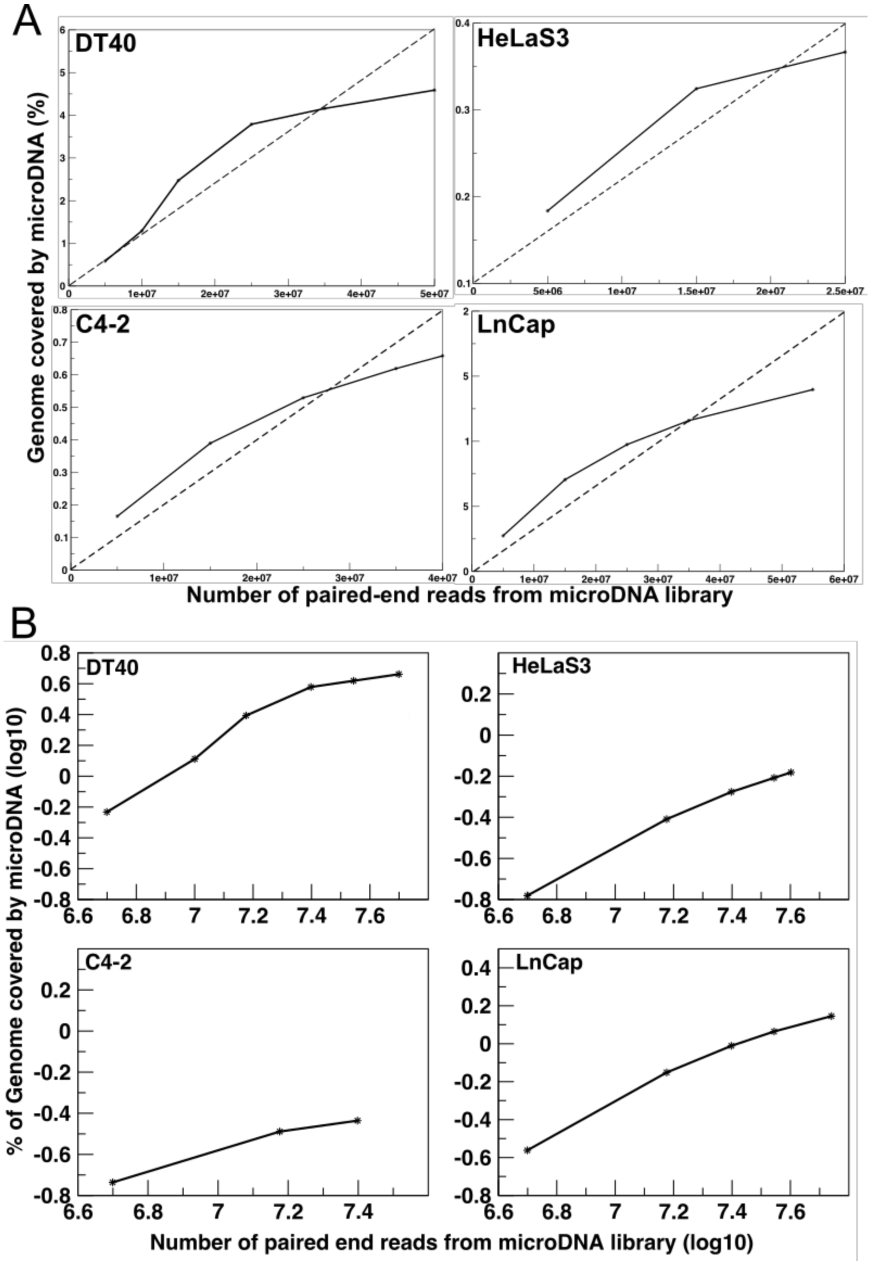

Supplementary Figure 5 Percentage of the genome that give rise to microDNA in indicated cell lines graphed on a (A) linear scale and (B) logarithmic scale. As we prepare many libraries and sequence them, the % of the genome covered by the microDNA sequences does not increase linearly but appears to saturate at 4-5 % of the genome for chicken DT40 cells, and 0.4-1.5% of the genome for human HeLa, C4-2, and LNCaP cells.

Name			Primer Sequence	MicroDNA sequence	Legend
miR145	AB	F	CCCCCCAGAGCAATAAGCC	CCCCCCAGAGCAATAAGCCACATCCGGCGACGTGTGGCACCCCACCCTGG<br>CTGCTACAGATGGGGCTGGATGCAGAAGAGAACTCCAGCTGGTCCTTAGG<br>GACACGGCGGCCTTGGCGCTGAAGGCCACTCGCTCC**CACCTTGTCCTCA**<br>**CGGTCCAGTTTTCCCAGGAATCCCTTAGATGCTAAGATGGGGATTCCTGG**<br>**AAATACTGTTCTTGAGGTCATGGTT**TCACAGCTGGATTTGCCTCCTTC	Bold: pre-hsa-mir-145 sequence<br>Underlined: 5p and 3p miR145 sequence
		R	GAAGGAGGCAAATCCAGCTGT		
	CD	F	GGCCACTCGCTCCCACCTTG		
		R	TTCAGCGCCAAGGCCGC		
miR191	AB	F	CCCCAGGAAGTAAGAGGGCTATCTTTAGCG	GCAGGAGCTCCCCCGCCCCCCGCCAA**CGGCTGGACAGCGGGCAACGGA**<br>**ATCCCAAAAGCAGCTGTTGTCTCCAGAGCATTCCAGCTGCGCTTGGATTT**<br>**CGTCCCCTGCTCTCCTGCCT**GAGCAGCGCCCTGGCCCAGATGGGGTGCC<br>CCTGACCCCCAGACATACTTTACTGAGCTGCTTGGGTCTCAGTTCCTCTCA<br>GTTGCGCCCTCAGGCTGGAGGTGATGGGTGTAGACGTGGGAGAGCCGAG<br>GCTGGTGGCCATCTCTGGAACCCTGGGGAGGATTGGCGAGGGAGGGTGG<br>ACCCAGGACCTCTGGGTAGGGCTGCAATGGTAGTGACTCCCCCAGGGCTG<br>CCTCAGCCCGCATCTCGCTAAAGATAGCCCTCTTACTTCCTGGGG	Bold: pre-hsa-mir-191 sequence<br>Underlined: 5p and 3p miR191 sequence
		R	GCAGGAGCTCCCCCGCCC		
	CD	F	AGAGGAACTGAGACCCAAGCAGC		
		R	CAGTTGCGCCCTCAGGCT		
miR126	AB	F	GGAGGATAGGTGGGTTCCCGA	GGAGGATAGGTGGGTTCCCGAGAACTGGGGGCAGGTTGCCCGGAGCCTC<br>ATATCAGCCAAGAAGGCAGAAGTGCCCCGTCCCGGGGTCCTGTCTGCATC<br>CAGCGCAGCATTCTGGAAGACGCCACGCCTC**CGCTGGCGACGGGACATT**<br>**ATTACTTTTGGTACGCGCTGTGACACTTCAAACTCGTACCGTGAGTAATAA**<br>**TGCGCCGTCCACGGCA**CCG	Bold: pre-hsa-mir-126 sequence<br>Underlined: 5p and 3p miR126 sequence
		R	CGGTGCCGTGGACGGCGCATT		
	CD	F	TTCTGGAAGACGCCACGCCTC		
		R	TGCTGCGCTGGATGCAGACAG		
Let-7	AB	F	TCCTCAGCCCTCTTTCCTCC	TCCTCAGCCCTCTTTCCTCCCGCGTCCCCAGGAGGTGCCTCTGGAAGCCA<br>CGGAGTCCCATCGGCACCAAGACCGACTGCCCTTTG**GGGTGAGGTAGTA**<br>**GGTTGTATAGTTTGGGGCTCTGCCCTGCTATGGGATAACTATACAATCTACT**<br>**GTCTTTCCT**GAAGTGGCTGTAATATCTGCGGTG	Bold: pre-hsa-let-7a sequence<br>Underlined: 5p and 3p let7a sequence
		R	CACCGCAGATATTACAGCCACTTC		
	CD	F	GGTAGTAGGTTGTATAGTTTGGG		
		R	CCAAAGGGCAGTCGGTCTT		
TESC-AS1	AB	F	TCTCCATCCAGTTGGGGTGACT	TCTCCATCCAGTTGGGGTGACTGGGGCCGATGCTGTTGCCTGTGTAATGTG<br>ATTCCTCCTCCTTAAAATAAG**GATAAGTTAATGAGATGTCCACCAGAGGGC**<br>**CTGGCGTGGACACAGCACATGGCCACAGAGGCTGGTGGGCGCTATGGAA**<br>**TCTTGTCCCCTGGAGAGGCACACAGCCAGGGCAGAACATCAAGGTCAAG**<br>**GCTCTCCTGAAGGCTCTGCAGTGCTTAGTGACACCACCATCAATGACCGT**<br>**CAGGTATCAG**GTCTGTTCTGAACGTTAGTCT	Bold: Portion of TESC-AS1 exon 2
		R	AGACTAACGTTCAGAACAGACCTGA		
	CD	F	CCCACCAGCCTCTGTGGC		
		R	CGCTATGGAATCTTGTCCCCTG		
SIGLEC9	AB	F	CAGGCTACATGCTGGCTGTG	CAGGCTACATGCTGGCTGTGGAGAGTCCACATCACTCAC**CTGAGAGGCTG**<br>**AACCCCTGACAGCGTTTGCATCCTCTATGCCCGTATCTCCCACGCCCGCT**<br>**GCTGGCCTTGCCGATTTCTTCCTGCAGGACCTCACT**CTGAGTGAAGAGAC<br>CAGAGAGCCTTTCAGTGTGGTCAGATTGG	Bold: Portion of SIGLEC9 exon 6
		R	CCAATCTGACCACACTGAAAGG		
	CD	F	CACGCCCGCTGCTGGCCT		
		R	GGAGATACGGGCATAGAGGATG		
KCNQ1OT1	AB	F	GAAAGGACACCATGTTGAACACATC	**ATCCCTTGTCAGATGAATATTTTCTCCCATTCTACAGGATATCTCTTAATAGT**<br>**TTATTATTTCCTTTTATGTGTAGGTTTTTAGTTTGATACAGTCCCATTTGTCTA**<br>**TTTTTGTTTTTGTTGCCTGTGCTTATAAAGTCTTACCCATAAAATCTTCGCTA**<br>**GACCCATATCCTGAAGGGTTTCCCTTCTGTTTTCTTCTAGTACTGTTTGCTT**<br>**CAGGTCTCATGTTTAAGTCTCTAATCAATTTTGAGTTGATTTTTTTATATGCT**<br>**GAGAGATAGTATAGTTTCATTCTTCTGCATATGATATCCTGTTTTCCCAACAT**<br>**GATGTGTTCAACATGGTGTCCTTTC**	Bold: Portion of KCNQ1OT1 exon 1
		R	ATCCCTTGTCAGATGAATATTTTCTCC		
	CD	F	AGGATATGGGTCTAGCGAAGATTTTATG		
		R	GAAGGGTTTCCCTTCTGTTTTCTTCTA		

Supplementary Table 1 Artificial microDNA were created using sequences of endogenous microDNA found in previous studies (Shibata 2012, Dillon 2015) which overlapped with microRNA.

The primer sequences amplified the staggered duplexes AB or CD for each microDNA. When these duplexes were annealed and subjected to LAMA we obtained the circular microDNA derived entirely from genomic sequence which matched the microDNA sequence acquired in previous studies. The sequences are listed under the ‘MicroDNA sequence’ column.
